# Association between choline supplementation and Alzheimer’s disease risk: a systematic review protocol

**DOI:** 10.3389/fnagi.2023.1242853

**Published:** 2023-08-28

**Authors:** Sixtus Aguree, Maryam Zolnoori, Thea Patricia Atwood, Arthur Owora

**Affiliations:** ^1^Department of Applied Health Science, School of Public Health, Indiana University Bloomington, Bloomington, IN, United States; ^2^Columbia University Irving Medical Center, New York, NY, United States; ^3^Department of Chemistry, Indiana University Bloomington, Bloomington, IN, United States; ^4^Department of Pediatrics, School of Medicine, Indiana University, Indianapolis, IN, United States

**Keywords:** one-carbon metabolism, dementia, nutrition and cognition, aging, geriatrics, nutrition and brain, acetylcholine

## Abstract

**Background and aims:**

There is growing evidence suggesting choline intake might have beneficial effects on cognitive function in the elderly. However, some studies report no relationship between choline intake and cognitive function or improvement in Alzheimer’s disease patients. This protocol is for a systematic review of choline intake and Alzheimer’s disease that aims to assess the comparative clinical effectiveness of choline supplementation on Alzheimer’s disease risk.

**Methods and analysis:**

literature search will be performed in PubMed, MEDLINE, EMBASE, CINAHL, Scopus, Cochrane, and the Web of Science electronic databases from inception until October 2023. We will follow the Preferred Reporting Items for Systematic Reviews and Meta-Analyses (PRISMA) guidelines. Studies will be included if they compared two different time points of choline biomarkers measures in men or women (65+) with Alzheimer’s Disease. The risk of bias in the included studies will be assessed within the Covidence data-management software.

**Results:**

This review will summarize the clinical trial and quasi-experimental evidence of choline intake on Alzheimer’s disease risk for adults aged 65+. The results from all eligible studies included in the analysis will be presented in tables, text, and figures. A descriptive synthesis will present the characteristics of included studies (e.g., age, sex of participants, type, length of intervention and comparator, and outcome measures), critical appraisal results, and descriptions of the main findings.

**Discussion:**

This systematic review will summarize the existing evidence on the association between Choline intake and AD and to make recommendations if appropriate. The results of this review will be considered with respect to whether there is enough evidence of benefit to merit a more definitive randomized controlled trial. The results will be disseminated through peer-reviewed journals population.

**Conclusion:**

This protocol outlines the methodology for a systematic review of choline intake and AD. The resulting systematic review from this protocol will form an evidence-based foundation to advance nutrition care for individuals with AD or poor cognitive function.

**Systematic review registration:**

http://www.crd.york.ac.uk/PROSPERO, identifier CRD42023395004.

## 1. Introduction

Alzheimer’s disease (AD) is a fatal neurodegenerative disorder characterized by cognitive decline, memory loss, behavioral changes, and other neurological symptoms (NIH, 2021). AD is the most common cause of dementia, accounting for 60% to 80% of cases (Alzheimer’s Association, 2023a). It is distinguished by anomalous protein deposits in the brain, such as beta-amyloid plaques and tau tangles (Alzheimer’s Association, 2023a). AD-related cognitive impairment in the US was estimated to affect 6.08 million Americans in 2017 and is projected to reach 15.0 million by 2,060 (Brookmeyer et al., 2018). Approximately 6.7 million American adults age 65+ are living with Alzheimer’s dementia in 2023, projected to reach 13.8 million by 2060 (Alzheimer’s Association, 2023a). AD is debilitating, affecting the patient’s quality of life and their families and creating a high financial burden on the healthcare system (Alzheimer’s Association, 2013, 2023a). Although the precise causes of Alzheimer’s disease are unknown, several risk factors have been identified, including aging and lifestyle factors such as diet (Smith et al., 2010; Alzheimer’s Association, 2013). Age is the strongest predictor or determinant of AD (Hebert et al., 2013)–AD is more common in the elderly over 65 (Alzheimer’s Association, 2011, 2023a; Hebert et al., 2013).

There are limited pharmacological interventions, such as Aducanumab for reduce amyloids levels and slight improvement in symptoms and Donepezil for treating memory and thinking difficulties in moderate-severe dementia (Cummings et al., 2021; Alzheimer’s Association, 2023b). These treatments are often accompanied by serious side effects, including amyloid-related imaging abnormalities, falls, and vomiting (Li et al., 2020). Due to these challenges, attention has turned to alternative approaches, specifically lifestyle modifications (Alzheimer’s Association, 2013; Martins et al., 2021). Of these, dietary supplements have become a primary focus, potentially holding promise in not just decelerating the progression from mild cognitive impairment to dementia but also delaying the advancement toward Alzheimer’s disease (Mosconi and McHugh, 2015; Gardener and Rainey-Smith, 2018; van den Brink et al., 2019). For instance, homocysteine-lowering B vitamins intakes are reported to protect against brain atrophy in the elderly, slowing down mental decline (Smith et al., 2010; Douaud et al., 2013; de Jager, 2014).

Another essential nutrient linked to regulating homocysteine level and brain function is choline. Choline is essential for maintaining the structural integrity of all cells, including brain cells (Hollenbeck, 2012) and studies have reported choline supplementation may benefit individuals with cognitive impairment or neurodegenerative diseases such as Alzheimer’s disease (Smith et al., 2010; Douaud et al., 2013; de Jager, 2014). One possible mechanism linking choline to improved neurocognition is its involvement in regulating one-carbon metabolism (Nurk et al., 2013), which is required for synthesizing phospholipids and other components of cell membranes essential for brain function. Choline is metabolized in the liver to generate a variety of metabolites, including betaine and phosphatidylcholine (Ueland et al., 2005). Betaine can donate a methyl group to homocysteine (an amino acid), generating S-adenosylmethionine (SAM), a critical methyl donor in one-carbon metabolism (Lu et al., 2001; Niculescu and Zeisel, 2002). SAM is an essential methyl donor in one-carbon metabolism and is involved in numerous cellular processes, including DNA and RNA methylation, which modulate gene expression, and neurotransmitter synthesis, which influences brain function (Lu et al., 2001; Bekdash, 2021).

Furthermore, choline is needed to produce acetylcholine, an important neurotransmitter for memory, mood, muscle control, and other brain and nervous system functions (National Academy of Sciences, 1998; Blusztajn et al., 2017). Loss of brain cell membrane function and intercellular communication is a hallmark of Alzheimer’s disease (Azam et al., 2021). Alzheimer’s disease patients are also reported to have decreased levels of the enzyme responsible for converting choline to acetylcholine in the brain (Higgins and Flicker, 2003) and phosphatidylcholine concentration (Whiley et al., 2014). As such, it has been suggested that consuming more phosphatidylcholines may slow the progression of Alzheimer’s disease (Higgins and Flicker, 2003). Phosphatidylcholine can serve as a phospholipid precursor; it may aid in maintaining the structural integrity of neurons, thereby promoting cognitive function in elderly/adults (Leermakers et al., 2015). Approximately 50 percent of the choline ingested in the United States is in the form of phosphatidylcholine (Sanders and Zeisel, 2007; Leermakers et al., 2015). Meat, poultry, fish, dairy products, and eggs are the principal dietary sources of choline in the United States, as they are particularly abundant in choline (Sanders and Zeisel, 2007; Chester et al., 2011; Hollenbeck, 2012; Leermakers et al., 2015).

Few observational studies have found an association between higher choline intakes and plasma concentrations and adult cognitive performance. In one observational study involving 2,195 adults aged 70–74 years in Norway, participants with plasma-free choline concentrations below 8.4 mcmol/L (20th percentile of concentrations in the study population) had poorer sensorimotor speed, perceptual speed, executive function, and global cognition than those with choline concentrations above 8.4 mcmol/L (Nurk et al., 2013). In another study involving 1,391 participants (aged 36–83) from the Framingham Offspring study, those with higher choline intake, as reported by food frequency questionnaires (between 1991–1995 and later 1998–2001), demonstrated enhanced verbal and visual memory (Poly et al., 2011).

Also, several small randomized intervention studies have demonstrated that choline supplements enhance adult cognitive performance (Buchman et al., 2001; Naber et al., 2015). Additional analysis of data (3,224 participants) from the Framingham Heart Study Offspring Cohort exams 5 to 9 revealed that a low choline intake was linked to a higher risk of developing dementia and Alzheimer’s disease (Yuan et al., 2022). These findings are consistent with observational studies showing that higher blood biomarkers of choline and betaine are associated with reduced risk of cognitive impairment in patients with acute ischemic stroke (Zhong et al., 2021). More compelling results linking Alzheimer’s disease and low choline intake, on the one hand, and the neuroprotective effect of supplementing choline in AD have been reported in animal models (Velazquez et al., 2019, 2020; Wang et al., 2019). Furthermore, recent studies reported an association between AD progression and low circulating choline levels in humans (Judd et al., 2023). In mice, dietary choline deficiency affected the function of the hippocampal network related to microtubules, regulation of the postsynaptic membrane, and the networks of proteins associated with mitochondrial function and inflammation (Dave et al., 2023). What is missing is evidence of choline’s effects compiled from pertinent studies. Some questions still remain unanswered, such as:(1) Based on the current body of research, to what extent do deficits contribute to an increased risk of Alzheimer’s disease? (2) To what degree are elevated choline levels (or blood choline concentrations) protective? (3) What is the minimum level of exposure necessary to experience a benefit? One review was conducted almost 10 years ago on the effect of choline and health across the life course. The review found that having higher blood metabolites of choline was associated with a lower risk of developing Alzheimer’s disease (Leermakers et al., 2015). But that review was not specific to Alzheimer’s disease, and there have since been additional studies on choline and Alzheimer’s disease in the elderly.

Systematic reviews are needed to elucidate the connection between choline consumption and Alzheimer’s disease and other forms of dementia. Therefore, we present a protocol of the methodology to undertake a systematic review, to evaluate population-based epidemiological associations between choline intake and Alzheimer’s disease in adults aged 65+ years.

## 2. Methods

### 2.1. Study registration

The protocol for this systematic review protocol has been registered with the Prospective Register of Systematic Reviews (PROSPERO) database (University of York, UK; http://www.crd.york.ac.uk/PROSPERO/: PROSPERO, registration number: CRD42023395004). The systematic review adheres to the Preferred Reporting Items for Systematic Reviews and Meta-analysis (PRIMSA) guidelines (Liberati et al., 2009; Moher et al., 2009). Modifications to the protocol will subsequently be reported in PROSPERO.

### 2.2. Search strategy and selection criteria

A specific search strategy will be developed by the principal investigators and reviewed by a Health Science Liberian with expertise in systematic review and meta-analysis. The initial search will be developed on PubMed-MEDLINE using varied Medical Subject Headings (MeSH) and free-text terms and then translated into electronic databases. The search will include combinations of keywords related to choline, Alzheimer’s disease or dementia, and aging in the article titles, abstracts, and keywords such as “Alzheimer’s disease,” “AD,” “dementia,” “cognitive function,” “cognitive impairment,” “cognitive decline,” “choline,” or “dimethylglycine” or “trimethylamine N-oxide” or “TMAO” or “Citicoline” or “cytidine 5’-diphosphocholine,” phosphatidylcholine.” The Boolean operators (“AND” and “OR”) will be applied to each term in every set. A comprehensive literature search will be performed on PubMed, Web of Sciences, PsycINFO, EMBASE, SCOPUS, and the Cochrane Library databases to identify relevant studies published between the inception of each database and October 2023. The queries will be re-run just before the final analyses, and additional studies will be retrieved for inclusion. Combinations of choline, Alzheimer’s disease or dementia, and aging-related keywords will be included in article titles, abstracts, and keywords. The search strategy for MEDLINE is displayed in Table 1, for example. The final report will detail the complete search strategy in an appendix. To obtain a more exhaustive retrieval, a manual search of the reference lists of each eligible study will be performed. All considered studies will be imported into reference management software (EndNote software, version 20, Clarivate), and duplicate publications will be deleted.

**TABLE 1:** Sample search strategy in MEDLINE.

Description	String
Population: elderly adults, Alzheimer’s disease Exposure/Intervention: Choline Outcome: cognitive function	(exp *aged/or exp *geriatrics/or exp *geriatric psychiatry/or exp *geriatric nursing/or exp *geriatric psychiatry/or exp *health services for the aged/or (elder* or eldest or frail* or geriatric* or old age* or oldest old* or senior* or senium or very old* or septuagenarian* or octagenarian* or octogenarian* or nonagenarian* or centenarian* or centenarian* or supercentenarian* or older people or older subject* or older patient* or older age* or older adult* or older man or older men or older male* or older woman or older women or older female* or older population* or older person*).ti, ab, oa, kw.). OR ((Exp Dementia/OR exp Alzheimer disease/OR exp cognitive dysfunction/OR exp cognition disorders/) OR (Alzheimer*.ti, ab, oa, kw.) OR (Alzhiemer*.ti, ab, oa, kw.) OR (cognitive decline.ti,ab,oa, kw.) OR (cognitive impairment.ti, ab, oa, kw.)) AND (Exp Choline/OR (cholin* or dimethylglycine or trimethylamine N-oxide or TMAO).ti, ab, oa, kw). AND Measures of Alzheimer’s disease outcomes The Alzheimer’s Disease Assessment Scale - Cognitive Subscale (ADAS-COG), the Mini-Mental State Exam (MMSE), and the Clinical Dementia Rating (CDR) scale sum of boxes (CDR-SB), Consortium to Establish a Registry for Alzheimer’s Disease (CERAD) word Learning subtest, Animal Fluency (AF) test, Digit Symbol Substitution test (DSST), PET and CSF imaging measures

### 2.3. Inclusion criteria

All AD-related studies meeting the following criteria will be included as eligible articles: (1) original research studies (observational, cross-sectional, case-control, longitudinal, and interventional designs with a control group); (2) human investigations conducted on older adults or the elderly (age 65 and older); (3) studies describing the consumption of dietary choline or choline supplement, or measuring blood biomarkers of choline or dietary or supplemental choline; (4) studies that provided information on the methodologies used to assess AD, dementia, and other cognition-related health outcomes such as cognitive impairment and cognitive decline; (5) reported risk estimates (relative risk or hazard ratios (HRs) and corresponding 95% confidence intervals (CIs) for dementia or AD, or provided enough information to calculate effect size; (6) reported dementia or AD incidence at follow-up, if longitudinal; and (7) studies written in English with entire available texts. The following criteria will be applied to exclude studies:(1) reviews and book chapters or secondary-research evidence such as meta-analysis; (2) non-individual studies such as ecological methods; (3) overlapping studies (the study with the smaller sample size); and (4) studies with incomplete data (in which relative risk or hazard ratios for dementia were not reported or the study was only published as an abstract).

All participants will be included in such investigations, regardless of age, gender, nationality, or inpatient or outpatient status.

### 2.4. Outcome measures

We will assess the association between choline and AD using data from RCTs, quasi-experimental studies, and observational. We have identified the preferred cognitive outcomes as changes in memory, executive functions, language and communication, judgment and insight, orientation, and spatial cognition. The outcome measure metrics will be measures of association (RR, ORs, regression coefficients, etc.) from these studies. The National Institute on Aging-Alzheimer’s Association (NIA-AA) (Albert et al., 2011; Jack et al., 2011; McKhann et al., 2011; Sperling et al., 2011) and the International Working Group (IWG) (Dubois et al., 2007, 2010, 2014) proposed diagnostic criteria for AD will be used. To be clear, we will consider AD to include both the underlying disease process (*pathophysiological*, e.g., Aβ amyloidosis) and the various clinical stages of the illness (Sperling et al., 2011). Studies should specifically include AD or ADRD patient population to be considered.

Advancements in neuroimaging [amyloid positron emission tomography (PET)] and cerebrospinal fluid (CSF) assays biomarkers allow for studying the preclinical state of Alzheimer’s pathology (AP) (Dubois et al., 2016) to detect evidence of Alzheimer’s disease *in vivo* (Pontecorvo and Mintun, 2011; Bateman et al., 2012; Johnson et al., 2013; Dubois et al., 2016). These advances have been incorporated into the new NIA-AA and IWG criteria for defining AD. Because NIA-AA and IWG are relatively new revision to National Institute of Neurological and Communicative Disorders and Stroke and the Alzheimer’s Disease and Related Disorders Association (NINCDS-ADRDA) criteria which has been in existence for about four decades McKhann et al. (1984), we expect most available studies to be based on NINCDS-ADRDA. Thus, studies where clinical diagnosis of AD was based on the NINCDS-ADRDA criteria will be accepted. The NINCDS-ADRDA approach involves evaluating cognitive and functional impairments through clinical assessments while ruling out other potential causes of dementia.

Thus, we would consider studies suitable for inclusion if they have reported a measurable impact of choline on cognitive function, such as improvement in cognitive assessment tests (e.g., performance-based assessments of cognitive functions such as the Alzheimer’s Disease Assessment Scale - Cognitive Subscale (ADAS-COG), the Mini-Mental State Exam (MMSE), and the Clinical Dementia Rating (CDR) scale sum of boxes (CDR-SB), Consortium to Establish a Registry for Alzheimer’s Disease (CERAD) word Learning subtest, Animal Fluency (AF) test, Digit Symbol Substitution test (DSST), PET and CSF imaging measures, or other AD biomarkers. Conversely, studies focusing solely on the impact of choline on sleep will be excluded from our review since they do not align with our defined outcomes. Cognitive assessment based on self-report will also be excluded as they have been shown to be unreliable.

### 2.5. Data collection and analysis

Pre-designed, standardized data extraction forms will be created to record pertinent information from each included study. Data extraction will be performed using Covidence software^1^ (Babineau, 2014; Covidence, 2020). All citations identified by our search strategy will be imported into Covidence systematic review software, where duplicates will automatically remove. Two reviewers will independently screen the studies for titles and abstracts against the inclusion and exclusion criteria. A third reviewer will solve conflicts and discrepancies that emerged during the two screening stages. The reference lists of eligible studies will also be screened to identify additional studies that might have been missed.

The data to be extracted from the eligible articles are the first author’s name, year of publication, the country where the study was conducted, study design, method of randomization, exposure or intervention details (which can include assessment method, distribution in the study population, and dosage when describing choline supplements), control group details, sample size, patient demographics, including age and sex, criteria used to diagnose AD, all outcome measures, follow-up duration, dietary intervention protocol, main results, distribution of results, statistical methods, and adjustment.

A final collation of data will be imported into STATA for statistical review and analysis. Each final article will be subjected to a quality and risk of bias assessment within Covidence. The Covidence software has a built-in Cochrane risk of bias assessment tool. We will use the Cochrane risk-of-bias tool for randomized trials (RoB 2) to assess the risk of bias in randomized trials and the ROBINS-I tool to assess non-randomized studies of interventions included in the study (Sterne et al., 2016, 2019). The GRADE (grading of recommendation, assessment, development, and evaluation) assessment will then be conducted to evaluate the included studies. The GRADE assessment determines the quality of evidence by considering factors such as study design, risk of biases, precision, consistency, directness, and other reported aspects. The evidence is then classified as very low, low, moderate, or high.

Statistical analysis will be performed using STATA software 17.0 (Stata Corporation, College Station, TX, USA) with the contributed “metan” (Harris et al., 2008), “metabias” (Harbord et al., 2009), and “confunnel” (Palmer et al., 2008; Peters et al., 2008) packages. Study heterogeneity will be assessed using *I*^2^ estimations. The *I*^2^ estimation is a statistical measure representing the percentage of variation across studies due to heterogeneity rather than chance. Using Cochrane review group criteria, study heterogeneity (Higgins et al., 2003) will be divided into three levels: low heterogeneity (*I*^2^ < 25%), moderate heterogeneity (*I*^2^ 25–50%), and high heterogeneity (*I*^2^ > 50%). Cochran Q statistics will be conducted to evaluate the heterogeneity between studies, where *P* < 0.10 will be regarded as statistically significant. A *P* > 0.10 in the Q test and *I*^2^ < 50% will indicate no heterogeneity. A formal meta-analysis will be conducted if deemed suitable based on the quantity and quality of eligible studies. We will use a fixed effects model if no statistical heterogeneity is detected; otherwise, a random effects model will be used. RR, ORs, and regression coefficients will be considered measures of effect size for eligible studies. We will use forest plots to depict the results graphically. Publication bias will be evaluated by the funnel plot and Begg’s test. Visually symmetrical distribution of data points (*p* > 0.05) will be considered as indicating no (low) publication bias. We will perform a leave-one-out analysis to assess the influence of each study. This sensitivity analysis evaluates each study’s influence on the overall pooled effect size by omitting one study at a time to examine the stability of the results. Subgroup analyses will consider age (<80 vs. >80), duration of intervention, study design (RCT vs. observational), and differences between men and women. A 2-tailed *P* < 0.05 will be considered statistically significant.

We will perform a narrative synthesis if there is a high heterogeneity (*I*^2^ > 50%) study, such as in study designs, exposure, outcome measures, and analytical methods that makes it inappropriate to statistically combine all the included studies in a meta-analysis. A formal narrative synthesis on quantitative studies will be undertaken according to the reporting guideline of the synthesis without meta-analysis (SWiM) (Campbell et al., 2020). The included studies will be grouped by study designs and ordered by publication years. Vote-counting methods based on directions of effect and *P*-values will be applied. Quality assessments on studies included will be considered when interpreting findings.

## 3. Discussion

This will be the first systematic evaluating of the effect of choline intake on AD. There is evidence that choline consumption might help improve cognitive performance or delay cognitive deterioration in the elderly, but a systematic review on this topic is currently lacking. The proposed systematic review will strengthen the evidence base on what is known regarding associations between choline intake and AD by identifying, evaluating, and synthesizing the findings of existing clinical and observational studies on this topic. We will conduct a structured literature search using the Medline, EMBASE, and Cochrane CENTRAL databases from inception to October 2023. Then formal inclusion and exclusion criteria will be applied to the review articles found via these searchers to obtain eligible studies for further evaluation. From the eligible articles, we will perform data extraction, validity assessment, and meta-analyses if appropriate. Appropriateness for meta-analysis will be based on the total number of eligible studies meeting quality control checks. If heterogeneity (e.g., *I*^2^ = 50%) is detected, subgroup/sensitivity analysis may be used to explore the possible sources. If meta-analysis is not feasible based on the quality studies, narrative reviews will be performed.

Our report will be divided into two sections. The first section will delve into patients suffering from MCI, including pathophysiological conditions such as Aβ amyloidosis and symptomatic and amnestic cases. The second section will focus on individuals who have been diagnosed with Alzheimer’s disease, with a particular emphasis on the clinical stage of the condition. The implications of this systematic review extend beyond the immediate research community. If our study suggests a potential benefit of choline in combating the effects of AD, it could catalyze the initiation of a larger, more definitive randomized controlled trial. This could potentially bring us closer to an effective dietary strategy for mitigating the impact of AD, a leading cause of functional impairment in the elderly population, thus having profound implications for public health and healthcare policy.

## Data availability statement

The original contributions presented in this study are included in the article/supplementary material, further inquiries can be directed to the corresponding author.

## Ethics statement

This study is exempt from ethics approval or consent procedures as it does not include identifiable human data.

## Author contributions

SA: conceptualization, methodology (search strategy for English databases, analysis tools), investigation, data curation, project administration, writing—original draft, and writing—review and editing. MZ: methodology (search strategy for English databases, analysis tools), investigation, data curation, and writing—review and editing. TA: methodology, validation, and writing—review and editing. AO: conceptualization, methodology, validation, supervision, and writing—review and editing. All authors contributed to the article and approved the final version.

## Footnotes

1
https://www.covidence.org
